# Isorhapontigenin Modulates SOX9/TOLLIP Expression to Attenuate Cell Apoptosis and Oxidative Stress in Paraquat-Induced Acute Kidney Injury

**DOI:** 10.1155/2022/3328623

**Published:** 2022-06-09

**Authors:** Qiang Zheng, Yuan Zhang, Xiaofeng Wang, Fudong Wang, Hongyu Zhao

**Affiliations:** Department of Emergency Medicine, Shengjing Hospital of China Medical University, Shenyang, Liaoning, China

## Abstract

Paraquat (PQ) is a widely used herbicide but can be lethal to humans. The kidney is vital for PQ elimination; therefore, explorations for therapeutic approaches for PQ-induced acute kidney injury (AKI) are of great significance. Here, the effects of a natural bioactive polyphenol isorhapontigenin (ISO) on PQ-AKI were investigated. *In vitro* experiments carried out in PQ-intoxicated rat renal tubular epithelial cells (NRK-52E) showed that ISO treatment inhibited PQ-induced cell apoptosis and oxidative stress, which was evidenced by the decreased proapoptotic proteins [cleaved caspase 3/9 and poly (ADP-ribose) polymerase (PARP)], the reduced oxidative stress indicators [reactive oxygen species (ROS), malondialdehyde (MDA), and lactate dehydrogenase (LDH) leakage], and the increased antioxidants [superoxide dismutase (SOD), nuclear factor E2-related factor 2 (NRF2), and oxygenase-1 (HO-1)]. Furthermore, 50 mg/kg ISO pretreatment before PQ administration significantly attenuated PQ-AKI in rats, as manifested by the improved renal tubule damage, the reduced serum and urine markers of kidney injury, and the inhibited cell apoptosis and oxidative stress in the renal cortex. Furthermore, expression of sex-determining region Y box 9 (SOX9) and Toll-interacting protein (TOLLIP) in NRK-52E cells and the renal cortex was significantly upregulated after ISO treatment. Overexpression of SOX9 increased TOLLIP transcription and attenuated PQ-induced apoptosis and oxidative stress, whereas knockdown of SOX9 impaired the protective effects of ISO on NRK-52E cells against PQ toxicity. In conclusion, the present study demonstrated that ISO modulated SOX9/TOLLIP expression to attenuate cell apoptosis and oxidative stress in PQ-AKI, suggesting the potential of ISO in treating PQ-poisoned patients.

## 1. Introduction

Paraquat (1,1′-dimethyl-4,4′-bipyridium dichloride; PQ) is a widely used organic herbicide. Due to the lack of specific antidotes, exposure to PQ can be lethal to humans, with a high case fatality rate of 42.7% after ingestion [1]. Accelerating PQ excretion through the kidney will reduce the PQ accumulation in other organs, but the increased accumulation of PQ within kidneys may enhance the PQ nephrotoxicity and lead to acute kidney injury (AKI) [2], and the occurrence of AKI has been reported to be associated with the mortality in poisoning [3]. Hence, attenuating PQ-induced nephrotoxicity is of great significance for improving the prognosis of PQ-poisoned patients.

PQ damages the intracellular electron transfer systems and promotes the formation of superoxide anion (O_2_^−^), singlet oxygen, and other reactive oxygen species (ROS), which ultimately lead to the consumption of cellular nicotinamide adenine dinucleotide phosphate (NADPH) and lipid peroxidation of cell membranes [4–6]. Therefore, studies of the therapeutic potential of antioxidant agents against PQ toxicity have attracted much attention [7–9]. Isorhapontigenin (trans-3,5,4′-trihydroxy-3′-methoxystilbene; ISO) is a natural bioactive polyphenol, present in various plants and fruits [10]. Accumulating evidence has revealed the pharmacological effects of ISO in cellular and animal models, including antitumor, anti-inflammatory, and antiapoptotic effects [11–14]. Additionally, in *in vitro* oxidative damage models, ISO has been found to inhibit the malondialdehyde (MDA; a product of lipid peroxidation) formation and to rescue the decrease of glutathione (GSH; an important antioxidant) contents, suggesting its antioxidative roles [13]. Furthermore, ISO has been reported to slightly alleviate the renal impairment in diabetic mice [15]. However, it remains unknown whether ISO displays protective effects on PQ-AKI.

Sex-determining region Y box 9 (SOX9) is a transcription factor that participates in several physiological and pathological processes, including cartilage development and tumorigenesis of various tumors [16–19]. SOX9 has also been shown to be a marker of renal progenitor cells, and activation of SOX9-positive stem cells promotes epithelial regeneration after AKI in mice [20, 21]. Furthermore, SOX9-mediated tubular epithelial cell proliferation has been elucidated to contribute to the attenuation of cisplatin nephrotoxicity [22]. These findings imply the strong potential of SOX9 to alleviate kidney injury and promote renal repair. More interestingly, we found putative SOX9-binding domains in the promotor of the Toll-interacting protein (TOLLIP), an inflammation-regulating factor that has been determined to exhibit protective effects on PQ-AKI and PQ-induced lung injury in our previous works [23, 24]. These findings suggest that SOX9 might alleviate AKI by regulating TOLLIP expression.

Here in the present study, we explored the effects of ISO on PQ-AKI, mainly focusing on its antiapoptotic and antioxidative roles in PQ-intoxicated renal tubular epithelial cells. And we further investigated the involvement of the SOX9/TOLLIP axis in the pharmacological effects of ISO.

## 2. Materials and Methods

### 2.1. Cell Culture and Treatments

The rat kidney epithelioid cell line NRK-52E was purchased from Procell Life Science&Technology Co., Ltd. (Wuhan, China) and grown in Dulbecco's modified Eagle's medium (DMEM; Gibco, Grand Island, NY, USA) supplemented with 5% fetal bovine serum (FBS; Tianhang Biological Science Co. Ltd., Zhejiang, China), in a humidified atmosphere with 5% CO_2_ at 37°C. NRK-52E cells were transduced with adenoviruses containing SOX9 shRNA, SOX9 cDNA, or their corresponding control to achieve the knockdown or overexpression of SOX9.

### 2.2. Cell Viability Assay

NRK-52E cells were treated with 0, 5, 10, 20, 40, and 80 *μ*M ISO (CAS: 32507-66-7; Aladdin Reagent, Shanghai, China) for 24 h and then subjected to cell count kit-8 (CCK-8) reagent (KeyGEN BioTECH, KGA317, Nanjing, China) incubation for 2 h at 37°C to determine the appropriate ISO concentration used *in vitro*. To determine the effects of ISO on PQ-induced cytotoxicity, NRK-52E cells were pretreated with a low (5 *μ*M) or high (10 *μ*M) dose of ISO for 30 min and then incubated with 300 *μ*M PQ (CAS: 1910-42-5; Aladdin Reagent) for 24 h. Subsequently, cell viability was detected using the CCK-8 reagent.

### 2.3. Protein Lysates and Western Blotting

Protein lysates of renal cortex and NRK-52E cells were prepared using a cell lysis buffer supplemented with phenylmethanesulfonyl fluoride (PMSF) purchased from Beyotime Biotechnology (Shanghai, China). The nuclear and cytosolic fractions were obtained using the nuclear and cytoplasmic protein extraction kit (Beyotime). Protein samples were resolved via sodium dodecyl sulphate-polyacrylamide gel electrophoresis (SDS-PAGE) and transferred to polyvinylidene difluoride (PVDF) membranes (Millipore, Billerica, MA, USA). Following the transfer, the membranes were blocked with 5% nonfat milk for one hour at room temperature. Subsequently, the membranes were incubated with the following primary antibodies: cleaved caspase 3 (1 : 1000, CST, #9661, Danvers, MA, USA), cleaved caspase 9 (1 : 1000, CST, #9507), cleaved poly (ADP-ribose) polymerase (PARP; 1 : 1000, CST, #9545), nuclear factor E2-related factor 2 (NRF2; 1 : 1000, ABclonal, A0674, Wuhan, China), heme oxygenase 1 (HO-1; 1 : 1000, Affinity, AF5393, Changzhou, China), SOX9 (1 : 1000, Affinity, AF6330), TOLLIP (1 : 1000, ABclonal, A2202), Histone H3 (1 : 2000, ABGENT, AM8433, San Diego, CA, USA), and *β*-actin (1 : 1000, Santa Cruz, sc-47778, Dallas, TX, USA) overnight at 4°C. The next day, the membranes were incubated with horseradish peroxidase-conjugated goat antimouse/rabbit secondary antibodies (1 : 5000) for 45 min at 37°C. Finally, the membranes were imaged using the chemiluminescence method.

### 2.4. Measurement of Oxidative Stress

To measure ROS levels, cells were incubated with 2′,7′-dichlorofluorescein diacetate (DCFH-DA) probes (Beyotime) for approximately 30 min and then were subjected to flow cytometry detection. The ROS in cells could oxidize DCFH to produce fluorescent 2′,7′-dichlorofluorescein (DCF). Therefore, the fluorescence intensity of DCF indicated ROS levels. The MDA content, superoxide dismutase (SOD) activity, and lactate dehydrogenase (LDH) leakage were determined using commercially available kits (MDA, Nanjing Jiancheng Bioengineering Institute, A003, Nanjing, China; LDH, Wanleibio, WLA072, Shenyang, China; SOD, Nanjing Jiancheng Bioengineering Institute, A001) per manufacturers' instructions.

### 2.5. Quantitative Real-Time PCR (qPCR) Assays

Total RNA was extracted from renal cortex tissues and NRK-52E cells. RNA concentrations were measured using the Thermo Scientific NanoDrop 2000 (Pittsburgh, PA, USA). The cDNAs were obtained from reverse transcription and used as templates for qPCR. The qPCR was performed on a quantitative real-time PCR system, using the 2x Taq PCR Master Mix (Solarbio, PC1150, Beijing, China) and SYBR Green (Solarbio, SY1020). The primer sequences were given as follows (5′-3′): HO-1 forward (Fwd), CGAAACAAGCAGAACCCA; HO-1 reverse (Rev), CACCAGCAGCTCAGGATG; SOX9 Fwd, GCACATCAAGACGGAGCAA; SOX9 Rev, AGGTGAAGGTGGAGTAGAGCC; TOLLIP Fwd, CAGCCTGTGGTTCTGATG; and TOLLIP Rev, TCTTTGTTCCCTCTTTGG. The comparative CT (2^−*ΔΔ*CT^) method was used to calculate relative changes in gene transcripts (*β*-actin served as a housekeeping gene control).

### 2.6. Dual-Luciferase Reporter Assay

The dual-luciferase reporter assay was conducted to explore the transactivation activities of SOX9 on TOLLIP promoters. In brief, NRK-52E cells were cotransfected with SOX9-overexpressing plasmids and luciferase reporter plasmids containing the promoter region of the TOLLIP gene using the Lipofectamine™ 3000 Transfection Reagent (Thermo Scientific, L300015). At 48 h after transfection, cells were lysed, and luciferase levels were quantified by using the dual-luciferase reporter gene assay kit (KeyGEN BioTECH, KGAF040), with Renilla luciferase being the control.

### 2.7. Measurement of Cell Apoptosis

Flow cytometry was performed to determine the percentage of apoptotic cells. In brief, cells were incubated with Annexin V-FITC and 7-amino-actinomycin D (7-AAD) solution (KeyGEN BioTECH) for 15 min at room temperature and subjected to flow cytometry detection. Hoechst staining and terminal deoxynucleotidyl transferase dUTP nick end labeling (TUNEL) staining were performed to evaluate the cell death by using commercially available kits per manufacturers' protocols (Hoechst, KeyGEN BioTECH, KGA212; TUNEL, Roche Diagnostics, 12156792910, Basel, Switzerland).

### 2.8. PQ-AKI Models

All animal experiments were performed in accordance with the ARRIVE guidelines and the Guide for the Care and Use of Laboratory Animals [25]. The animal experiment protocols were approved by the Ethics Committee of Shengjing Hospital of China Medical University (accession number, 2020PS702K). PQ-AKI models were established according to previously reported research, in which the rats showed significant histological features and biomarkers of AKI after PQ injection [23, 26]. PQ was dissolved in normal saline to prepare the stock solution (625 mg/ml). Six- to eight-week-old male Wistar rats were used for establishing the PQ-AKI model. Rats were injected intraperitoneally with PQ (25 mg/kg; PQ working solution: 6.25 mg/ml) to induce AKI. ISO was intraperitoneally injected into rats at low (25 mg/kg) or high (50 mg/kg) doses once a day for a continuous seven days prior to PQ injection. Serum, urine, and kidney tissue samples were collected 24 h after injection of PQ. The metabolism cages were used to collect 24 h urine samples after PQ injection.

### 2.9. Serum and Urine Biochemistry Measurements

Serum and urine samples were collected 24 h after injection of PQ. Serum creatinine (Scr), blood urea nitrogen (BUN), urine neutrophil gelatinase-associated lipocalin (NGAL), and urinary proteins were measured using the following commercially available kits: Creatinine (Cr) Assay Kit (Nanjing Jiancheng Bioengineering Institute), Urea Assay Kit (Nanjing Jiancheng Bioengineering Institute), Rat Lipocalin-2/NGAL ELISA Kit (FineTest, Wuhan, China), and Urine Protein Test Kit (Nanjing Jiancheng Bioengineering Institute).

### 2.10. Renal Histological Analysis and Immunohistochemistry

Histological alterations in the kidney were evaluated after PQ injection. The tissues of the renal cortex of rats were embedded in paraffin, dissected into 5 *μ*m sections, and subjected to hematoxylin and eosin (H&E) staining and periodic acid-Schiff (PAS) staining. For determining the expression of SOX9 and TOLLIP in the renal cortex, the sections were incubated with the antibodies against SOX9 (Affinity, AF6330) or TOLLIP (A2202, ABclonal) overnight at 4°C. The next day, sections were incubated with the horseradish peroxidase-conjugated goat antirabbit secondary antibody for one hour at 37°C followed by incubation with diaminobenzidine (DAB; Maixin Biotech, Fuzhou, China) for color development. The representative images of sections were captured under the microscope at 100 and 400 magnifications.

### 2.11. Statistical Analysis

GraphPad Prism software (Version 9.0) was used for data analysis. Data were presented as means and standard derivations. When comparing variables between more than two groups, analysis of variance (ANOVA) was used for parametric data. Statistical significance was considered at *P* < 0.05.

## 3. Results

### 3.1. ISO Alleviates PQ-Induced Cell Apoptosis

**Figure 1:**
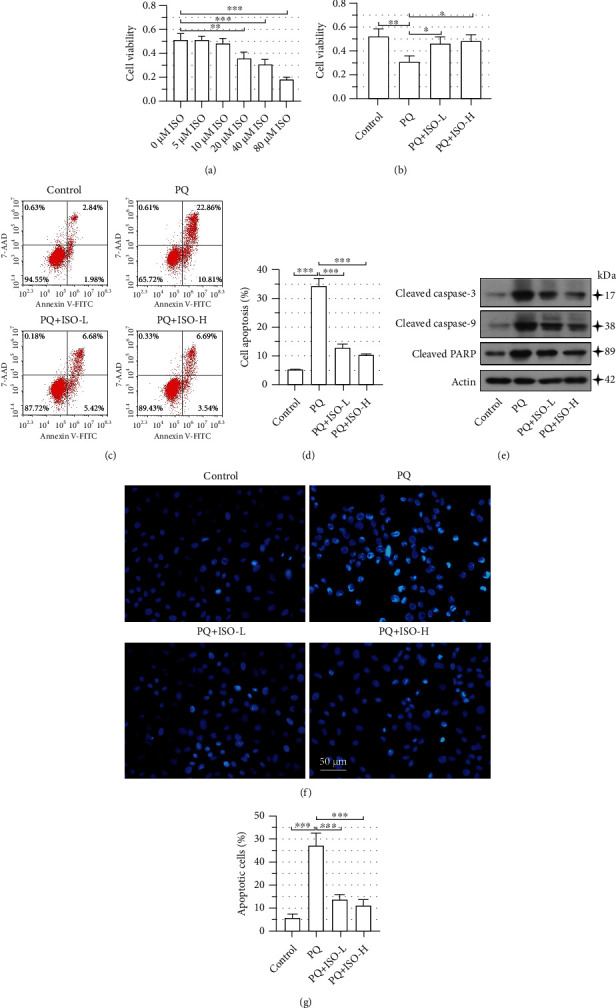
Isorhapontigenin (ISO) alleviates paraquat- (PQ-) induced cell apoptosis. CCK-8 assays showed the cell viability of NRK-52E cells incubated with (a) different concentrations of ISO for 24 h or (b) ISO (5 *μ*M or 10 *μ*M) for 30 min followed by a 24 h coincubation with 300 *μ*M PQ. (c, d) Flow cytometry analysis was performed to determine the percentage of apoptotic cells. (e) Western blot showed the expression of apoptosis-related proteins. (f) Cell death was analyzed via nuclear staining with Hoechst. (g) Quantitative analysis for apoptotic cells. The scale bar represents 50 *μ*m; 400x magnification. Error bars represent standard deviations. ^∗^*P* values < 0.05, ^∗∗^*P* values < 0.01, and ^∗∗∗^*P* values < 0.001.

As determined by the CCK-8 assay, 5 *μ*M and 10 *μ*M ISO had no obvious impact on the cell viability of NRK-52E cells (Figure 1(a)). Therefore, we used 5 *μ*M and 10 *μ*M ISO (ISO-L and ISO-H) to explore the effects of ISO on PQ-induced cell death. We found that pretreatment with ISO markedly reversed the cell viability reduced by PQ exposure (Figure 1(b)). Consistently, flow cytometry analysis showed that ISO significantly decreased the percentage of apoptotic cells after PQ incubation (Figures 1(c) and 1(d)). Similar results were obtained from the Hoechst staining (Figures 1(f) and 1(g)). Furthermore, as demonstrated by the Western blot analysis, the levels of proapoptotic proteins (cleaved caspase 3, caspase 9, and PARP) were markedly elevated in PQ-intoxicated cells, which was restored by ISO pretreatment (Figure 1(e)). These results indicated that ISO significantly alleviated the PQ-induced cell death in rat renal tubular epithelial cells.

### 3.2. ISO Ameliorates the PQ-Induced Oxidative Stress in NRK-52E Cells

**Figure 2:**
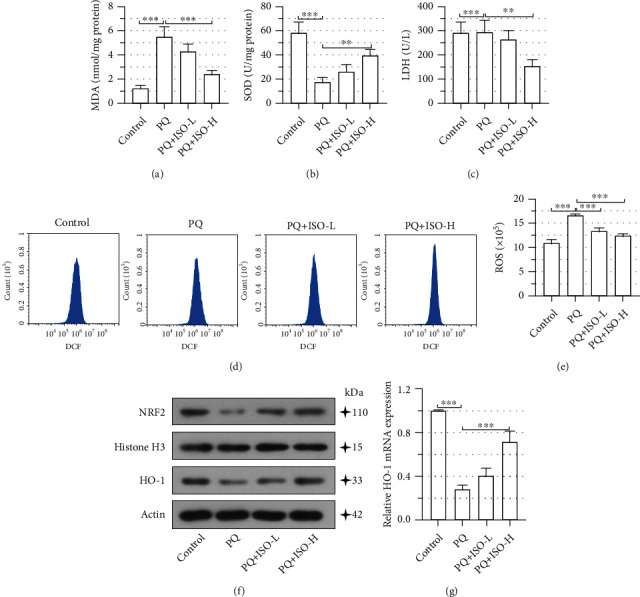
Isorhapontigenin (ISO) ameliorates the paraquat- (PQ-) induced oxidative stress in NRK-52E cells. NRK-52E cells were pretreated with ISO (5 *μ*M or 10 *μ*M) for 30 min followed by a 24 h coincubation with 300 *μ*M PQ. (a) The malondialdehyde (MDA) contents, (b) superoxide dismutase (SOD) activity, and (c) lactate dehydrogenase (LDH) leakage were determined using the commercially available kits. (d, e) Flow cytometry was performed to determine the reactive oxygen species (ROS) production in cells. The fluorescence intensity of 2′,7′-dichlorofluorescein (DCF) indicated ROS levels. (f) Protein levels of nuclear factor erythroid 2-related factor 2 (NRF2) and heme oxygenase-1 (HO-1) were detected via Western blot. (g) The mRNA levels of HO-1 were measured via quantitative real-time PCR. Error bars represent standard deviations. ^∗∗^*P* values < 0.01; ^∗∗∗^*P* values < 0.001.

Next, we evaluated the effects of ISO on PQ-induced oxidative stress. In PQ-intoxicated NRK-52E cells, MDA, a major toxic product of lipid peroxidation, was significantly increased, along with a significant decline in the activity of SOD and an obvious increase in the leakage of LDH (Figures 2(a)–2(c)). However, 10 *μ*m ISO pretreatment significantly protected cells from PQ-induced alterations (Figures 2(a)–2(c)). The flow cytometry analysis also determined that ISO pretreatment decreased the PQ-induced ROS production in NRK-52E cells (Figures 2(d) and 2(e)). Furthermore, Western blot and qPCR revealed that the upregulated expression levels of antioxidative molecules HO-1 and nuclear NRF2 were reversed by 10 *μ*m ISO pretreatment (Figures 2(f) and 2(g)). These findings supported that ISO ameliorated PQ-induced oxidative stress in NRK-52E cells.

### 3.3. SOX9 Regulates TOLLIP Expression and Attenuates PQ Toxicity to NRK-52E Cells

**Figure 3:**
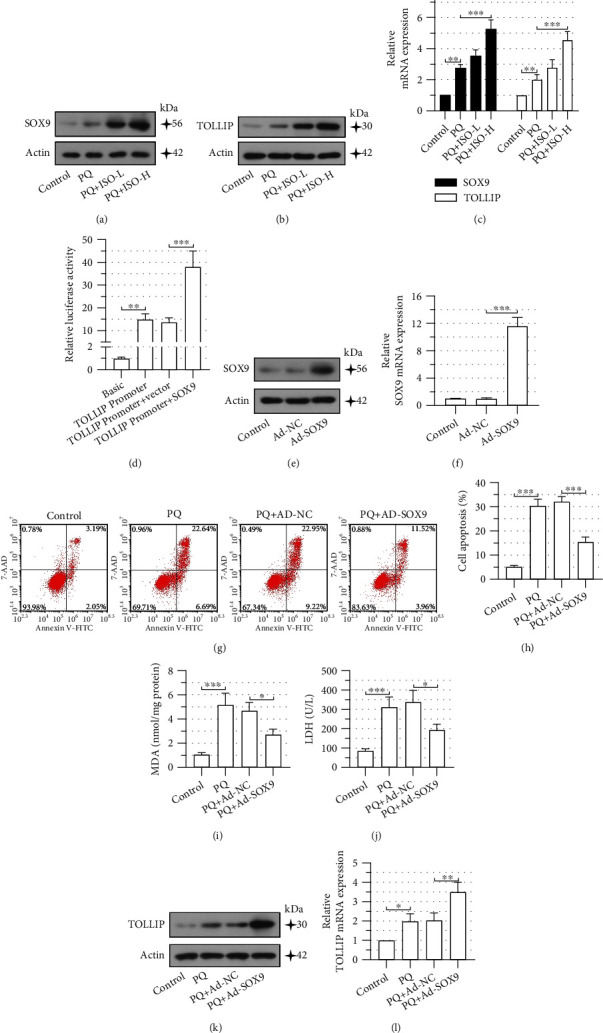
SRY-box transcription factor 9 (SOX9) regulates Toll-interacting protein (TOLLIP) expression and attenuates paraquat (PQ) toxicity to NRK-52E cells. NRK-52E cells were pretreated with ISO (5 *μ*M or 10 *μ*M) for 30 min followed by a 24 h coincubation with 300 *μ*M PQ. The expression of SOX9 and TOLLIP was evaluated at (a, b) protein and (c) mRNA levels using Western blot and quantitative real-time PCR (qPCR). (d) A dual-luciferase reporter assay was performed to explore the effects of SOX9 on the activities of the TOLLIP promoter. (e, f) Verification of adenovirus- (Ad-) mediated overexpression of SOX9 in NRK-52E cells using Western blot and qPCR. Forty-eight hours after Ad transduction, cells were incubated with 300 *μ*M PQ for 24 h. (g, h) Flow cytometry was performed to determine the cell apoptosis. Levels of (i) malondialdehyde (MDA) and (j) lactate dehydrogenase (LDH) were measured using the commercially available kits. TOLLIP expression was determined at the (k) protein and (l) mRNA levels using Western blot and qPCR. Error bars represent standard deviations. ^∗^*P* values < 0.05, ^∗∗^*P* values < 0.01, and ^∗∗∗^*P* values < 0.001.

Afterward, we investigated the role of SOX9 in PQ-intoxicated NRK-52E cells. As shown in Figures 3(a)–3(c), the upregulation of mRNA and protein levels of SOX9 and TOLLIP was observed in PQ-intoxicated cells and was enhanced by 10 *μ*m ISO pretreatment. And the dual-luciferase reporter assay validated the transcriptional regulation of TOLLIP by SOX9 (Figure 3(d)). Next, we overexpressed SOX9 in NRK-52E cells (Figures 3(e) and 3(f)) and found that PQ-induced cell apoptosis was significantly reduced in SOX9-overexpressing cells in comparison to control cells (Figures 3(g) and 3(h)), along with an obvious reduction in MDA production and LDH leakage (Figures 3(i) and 3(j)). Furthermore, overexpression of SOX9 upregulated TOLLIP expression in PQ-treated NRK-52E cells (Figures 3(k) and 3(l)). The above results suggested that SOX9 increased TOLLIP expression and attenuated PQ toxicity to NRK-52E cells.

### 3.4. SOX9 and TOLLIP Participate in the Pharmacological Effects of ISO

**Figure 4:**
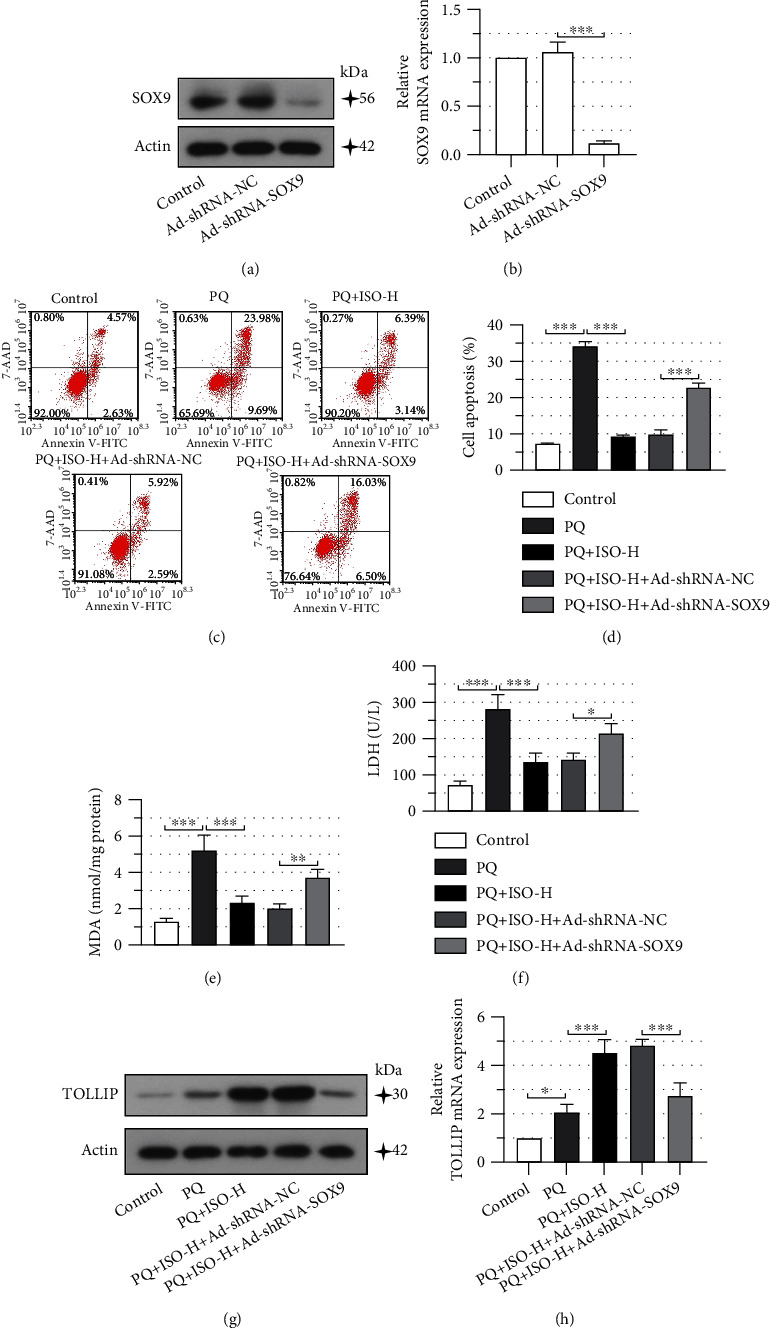
SRY-box transcription factor 9 (SOX9) and Toll-interacting protein (TOLLIP) participate in the pharmacological effects of isorhapontigenin (ISO). (a, b) Verification of adenovirus- (Ad-) mediated knockdown of SOX9 in NRK-52E cells using Western blot and quantitative real-time PCR (qPCR). Forty-eight hours after Ad transduction, cells were incubated with 300 *μ*M PQ for 24 h. (c, d) Flow cytometry was performed to determine the cell apoptosis. Levels of (e) malondialdehyde (MDA) and (f) lactate dehydrogenase (LDH) were measured using commercially available kits. TOLLIP expression was determined at the (g) protein and (h) mRNA levels using Western blot and qPCR. Error bars represent standard deviations. ^∗^*P* values < 0.05, ^∗∗^*P* values < 0.01, and ^∗∗∗^*P* values < 0.001.

Next, we performed the adenovirus-based knockdown of SOX9 in NRK-52E cells (Figures 4(a) and 4(b)). Downregulation of SOX9 impaired the ISO treatment-mediated inhibition of PQ-induced cell apoptosis (Figures 4(c) and 4(d)) and reversed the protective impacts of ISO on PQ-induced oxidative stress, as evidenced by the increased MDA and LDH, compared to the negative control adenovirus transduced cells (Figures 4(e) and 4(f)). Furthermore, the silencing of SOX9 markedly reduced the ISO-elevated TOLLIP expression in PQ-treated cells (Figures 4(g) and 4(h)). These results indicated that ISO might attenuate PQ-induced cell apoptosis and oxidative stress by regulating SOX9 and TOLLIP expression.

### 3.5. ISO Attenuates Renal Injury in PQ-AKI Rats

**Figure 5:**
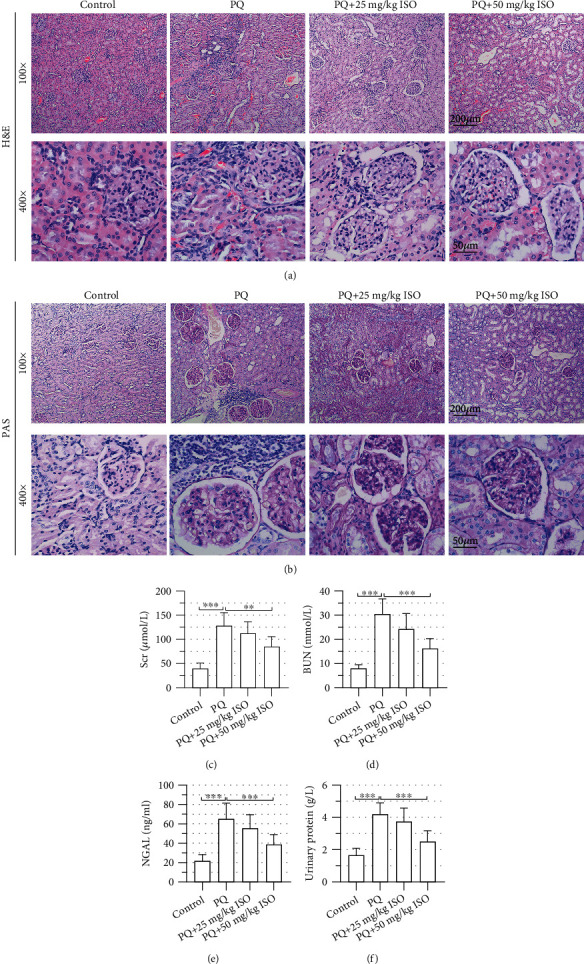
Isorhapontigenin (ISO) attenuates renal injury in paraquat- (PQ-) acute kidney injury (AKI) rats. Rats received an intraperitoneal injection of 25 mg/kg or 50 mg/kg ISO once a day for 7 days before injection of PQ. (a) Hematoxylin and eosin (H&E) staining and (b) periodic acid-Schiff (PAS) staining showed pathological alterations in the renal cortex of rats. Scale bars represent 200 *μ*m or 50 *μ*m; 100x or 400x magnification. (c) Serum creatinine (Scr), (d) blood urea nitrogen (BUN), (e) urinary neutrophil gelatinase-associated lipocalin (NGAL), and (f) urinary proteins were evaluated using commercially available detection kits. Eight rats in each group. Error bars represent standard deviations. ^∗∗^*P* values < 0.01; ^∗∗∗^*P* values < 0.001.

We further evaluated the protective effects of ISO *in vivo*. As determined by H&E staining and PAS staining, 50 mg/kg ISO significantly attenuated the damage of renal tubules in PQ-injected rats (Figures 5(a) and 5(b)). Furthermore, Scr, BUN, NGAL, and urinary proteins were obviously elevated after PQ intoxication but were restored via 50 mg/kg ISO treatment (Figures 5(c)–5(f)), suggesting that ISO preserves renal functions of PQ-AKI rats.

### 3.6. ISO Alleviates Cell Apoptosis and Oxidative Stress in the Renal Cortex of PQ-AKI Rats

**Figure 6:**
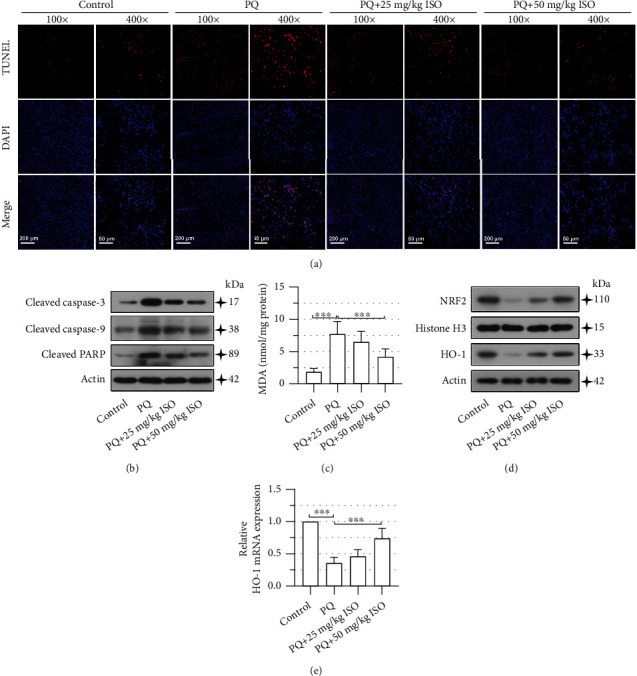
Isorhapontigenin (ISO) alleviates cell apoptosis and oxidative stress in the renal cortex of paraquat- (PQ-) acute kidney injury (AKI) rats. (a) Terminal deoxynucleotidyl transferase dUTP nick end labeling (TUNEL) staining showed the apoptotic cells in the renal cortex. Scale bars represent 200 *μ*m or 50 *μ*m; 100x or 400x magnification. (b) Expression of cell apoptosis-related proteins in the renal cortex was detected using Western blot. (c) Malondialdehyde (MDA) content in the renal cortex was measured using the commercially available kit. (d) Protein levels of nuclear factor erythroid 2-related factor 2 (NRF2) and heme oxygenase-1 (HO-1) were detected using Western blot. (e) The mRNA levels of HO-1 were measured using quantitative real-time PCR (qPCR). Eight rats in each group. Error bars represent standard deviations. ^∗∗∗^*P* values < 0.001.

TUNEL staining showed that cell apoptosis increased significantly in the renal cortex of PQ-AKI rats compared to control rats, but ISO reduced the number of apoptotic cells (Figure 6(a)). Consistently, the Western blot assay revealed that ISO treatment partially reversed the elevated levels of proapoptotic proteins (cleaved caspase 3, cleaved caspase 9, and cleaved PARP) in the renal cortex of PQ-AKI rats (Figure 6(b)). Furthermore, 50 mg/kg ISO markedly reduced the contents of MDA in the renal cortex of PQ-AKI rats (Figure 6(c)) and rescued the expression levels of HO-1 and nuclear NRF2 (Figures 6(d) and 6(e)). These results confirmed the protective impact of ISO on PQ-induced nephrotoxicity.

### 3.7. ISO Upregulates Expression Levels of SOX9 and TOLLIP in the Renal Cortex of PQ-AKI Rats

**Figure 7:**
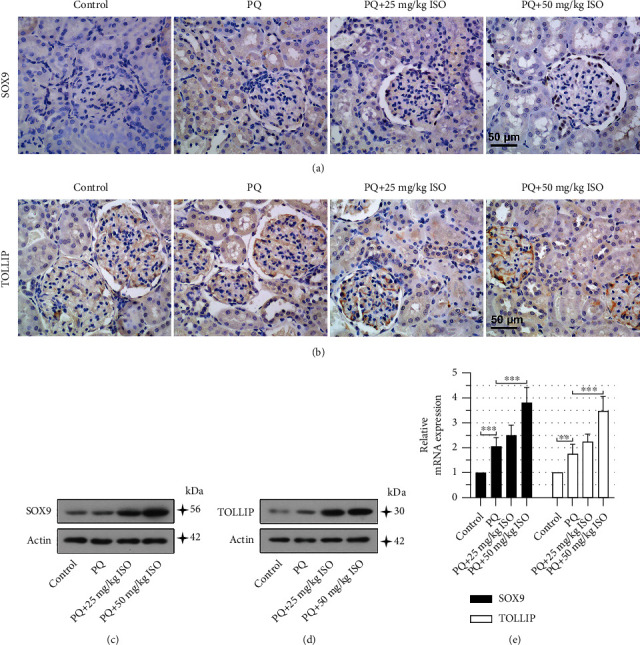
Isorhapontigenin (ISO) upregulates expression levels of SRY-box transcription factor 9 (SOX9) and Toll-interacting protein (TOLLIP) in the renal cortex of paraquat- (PQ-) acute kidney injury (AKI) rats. Immunohistochemistry staining for (a) SOX9 and (b) TOLLIP in the renal cortex. The scale bar represents 50 *μ*m; 400x magnification. Expression levels of SOX9 and TOLLIP in the renal cortex were determined at (c, d) protein and (e) mRNA levels using Western blot and quantitative real-time PCR (qPCR). Error bars represent standard deviations. Eight rats in each group. ^∗∗^*P* values < 0.01; ^∗∗∗^*P* values < 0.001.

Next, we evaluated the effects of ISO on the expression of SOX9 and TOLLIP in the renal cortex of PQ-AKI rats. Immunohistochemical staining revealed that SOX9 and TOLLIP were upregulated in the renal cortex of PQ-AKI rats and were further increased by ISO administration (Figures 7(a) and 7(b)). Western blot analysis and qPCR showed similar results (Figures 7(c)–7(e)). These findings implied the involvement of SOX9 and TOLLIP in the protective effects of ISO against PQ-AKI.

## 4. Discussion

In this study, we identified the therapeutic effects of ISO on PQ-induced AKI in rats. We also uncovered the molecular mechanism underlying the protective impacts, that ISO modulated SOX9/TOLLIP expression to alleviate PQ-induced cell apoptosis and oxidative stress in renal tubular epithelial cells. Our findings demonstrated the potential of ISO to attenuate PQ-induced nephrotoxicity.

AKI is a complex disorder, manifesting with an abrupt decline in renal filtration functions [27]. In PQ-poisoned patients, the incidence of AKI could reach 71.7% [28]. And several renal function indexes were used to predict PQ clearance and the prognosis of patients [3]. For example, Zhang et al. observed a negative correlation between the BUN and the ratio of urine-to-plasma PQ (an indicator to evaluate the PQ elimination) [29]. In addition, Scr showed strong power for evaluating the prognosis of acute PQ-poisoning patients and was identified as one of the independent risk indicators of in-hospital death events [30–32]. Furthermore, urinary NGAL, a sensitive marker to reflect the kidney injury [33], also displayed high sensitivity and specificity when predicting the mortality of acute PQ-poisoned patients [28, 34]. In previous research, ISO was reported to slightly alleviate the renal impairment in diabetic mice [15]. Consistently, we observed an obvious increase in these kidney injury indexes in PQ-AKI rats, while a high dose of ISO pretreatment significantly restored this increase, suggesting ISO-mediated protection of kidney functions after acute exposure to PQ.

It was reported that PQ could induce apoptosis of renal tubular epithelial cells to exsert nephrotoxicity [35, 36]. For example, PQ could modulate the expression of Bcl-2 family members such as proapoptotic Bax and antiapoptotic Bcl-2 to activate the intrinsic mitochondrial apoptotic pathway [7], as they could control the outer mitochondrial membrane permeability, thus regulating the release of apoptotic mediators [37]. Additionally, PQ could increase the generation of ROS and directly lead to mitochondrial outer membrane permeabilization, thus enhancing the release of cytochrome C from mitochondria into the cytosol. Cytosolic cytochrome C further binds to the apoptotic protease activating factor 1, contributing to the activation of the initiator caspase 9 and the executioner caspases like caspase 3 [38, 39].

Previous research demonstrated that ISO displayed antiapoptotic impacts on doxorubicin-induced cardiotoxicity and reduced the cleaved caspase 3 levels [14]. Consistently, in the present study, we found that ISO markedly decreased the PQ-mediated induction of active caspases 3 and 9, as well as the cleavage of PARP, a specific caspase substrate that prevents DNA repair-induced survival [40]. Furthermore, ISO was found to suppress the neuron apoptosis in cerebral ischemia/reperfusion injuries in rats, by decreasing the Bax and increasing the Bcl-2 [12]. These findings indicated that ISO effectively protected renal tubular epithelial cells from PQ-induced cell apoptosis, and this protection might be attributed to its modulation of Bax and Bcl-2 to restrain the mitochondrial outer membrane permeabilization.

Elevated ROS production during the redox cycle process is one of the main causes of PQ toxicity, which contributes to mitochondrial oxidative stress and cell death [4–6]. Oxidative stress is manifested by increased oxidative products and reduced antioxidants [41]. Normal cells have the ability to eliminate the excess ROS through antioxidant enzymes to prevent oxidative damage and maintain the oxidative balance [42]. However, severe exposure to toxicants such as PQ could harm the oxidative balance [43, 44]. For example, PQ significantly increased ROS and MDA production and LDH leakage but reduced the levels of the antioxidant enzyme SOD and the antioxidative molecules NRF2 and HO-1 [45, 46]. Therefore, antioxidants appeared to be promising approaches to relieve PQ-induced toxicity.

ISO was previously found to exhibit antioxidative effects by preventing the MDA production and antioxidant GSH reduction, and its antioxidative effects were much more potent than those of the classical antioxidant vitamin E [13]. Additionally, accumulating evidence revealed the mechanism underlying its antioxidative functions. First, ISO contains a very important active site (A4-hydroxyl group), which contributes to its activity to directly scavenge two classic ROS (hydroxyl and hydroperoxyl radical) [47]. Besides this, ISO could bind the flavin adenine dinucleotide site in xanthine oxidase (XO), to suppress the O_2_^−^ generation catalyzed by XO [48]. Given that XO played an important role in PQ toxicity, and the XO inhibitor significantly reduced the mortality of PQ-intoxicated rats [49, 50], ISO might also exhibit therapeutic effects on PQ-AKI by inhibiting XO. Furthermore, ISO was demonstrated to trigger the activation of NRF2 by increasing NRF2 expression and enhancing its nuclear translocation [51–53], thus significantly increasing the expression of various antioxidant enzymes. Compared to the direct scavenging for ROS or XO inhibition, the activation of NRF2 can amplify the antioxidant effect of ISO and prolong the duration of its functions.

Consistently, in our present study, we found that ISO pretreatment markedly increased SOD, NRF2, and HO-1 and reduced ROS, MDA, and LDH to protect against PQ-induced oxidative stress, supporting the beneficial role of ISO in maintaining the oxidative balance in PQ-intoxicated NRK-52E cells. Similar effects were observed in other cell types. For instance, ISO pretreatment also reduced intracellular ROS levels in airway epithelial cells in patients with chronic obstructive pulmonary disease [11], suggesting the potential therapeutic effects of ISO on PQ-induced lung injury as well.

The beneficial impacts of TOLLIP on PQ-AKI were described in our previous research. Specifically, TOLLIP suppressed TLR2/4-NF-*κ*B signaling to protect against PQ-induced NLRP3 inflammasome activation, thus attenuating kidney injuries [23]. Additionally, TOLLIP significantly antagonized bleomycin-induced caspase 3 protein cleavage and mitochondrial ROS accumulation to protect epithelial cells from apoptosis [54]. Conversely, TOLLIP exhaustion abolished the therapeutic effect of isorhynchophylline (a bioactive alkaloid) on PQ-induced oxidative stress and mitochondrial damage in NRK-52E cells [55]. Hence, TOLLIP might participate in the antiapoptotic and antioxidative functions of ISO.

SOX9 was reported to inhibit the apoptosis of chondrocytes [56] and was identified as an important marker in kidney repair, particularly in renal tubular epithelial regeneration [20]. Here in the present study, we reported the SOX9-mediated transcriptional regulation of TOLLIP and further determined that the regulation of SOX/TOLLIP expression was underlying the molecular mechanism of ISO effects on PQ-AKI. Interestingly, in a recent study, ISO protected against doxorubicin-induced cardiotoxicity via increasing the expression of YAP1 [14], and YAP1 could mediate the upregulation of SOX9 levels [57]. Therefore, the upregulated SOX9 might contribute to the ISO-mediated attenuation of kidney injuries and preservation of renal functions, and YAP1 might be involved in the ISO-mediated modulation of the SOX9 expression, but more experimental results were required for uncovering the molecular traits of the pharmacological effects of ISO.

## 5. Conclusions

In summary, our work uncovered the therapeutic effects of a natural bioactive polyphenol ISO on PQ-induced AKI and demonstrated that ISO modulated SOX9/TOLLIP expression to attenuate PQ-induced cell apoptosis and oxidative stress.

## Data Availability

The data that support the findings of this study are available from the corresponding author upon reasonable request.

## Supplementary Materials

Supplementary Materials Graphical abstract. Isorhapontigenin (ISO) pretreatment attenuates paraquat- (PQ-) induced acute kidney injury in rats. Mechanically, ISO prevents PQ-induced cell apoptosis and oxidative stress by upregulating SOX9 and TOLLIP expression.
