# Supplementary figures and images for: Isorhapontigenin Modulates SOX9/TOLLIP Expression to Attenuate Cell Apoptosis and Oxidative Stress in Paraquat-Induced Acute Kidney Injury
Source: Oxid Med Cell Longev. 2022 Jun 9;2022:3328623. doi: 10.1155/2022/3328623 (PMC9203234; doi:10.1155/2022/3328623)

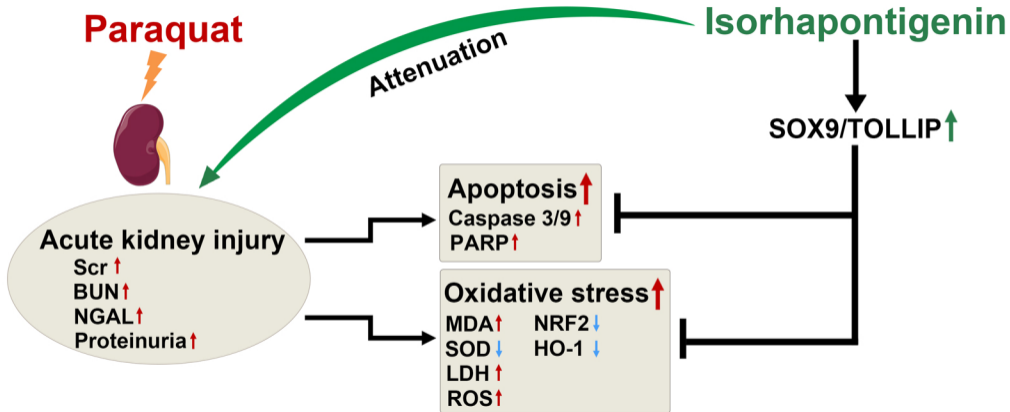

Supplement: Supplementary Materials — Graphical abstract. Isorhapontigenin (ISO) pretreatment attenuates paraquat- (PQ-) induced acute kidney injury in rats. Mechanically, ISO prevents PQ-induced cell apoptosis and oxidative stress by upregulating SOX9 and TOLLIP expression. [file 3328623.f1.pdf]
